# Albuterol Use in Children Hospitalized with Human Metapneumovirus Respiratory Infection

**DOI:** 10.1155/2016/7021943

**Published:** 2016-01-27

**Authors:** Lindsey K. Rasmussen, Jennifer Schuette, Michael C. Spaeder

**Affiliations:** ^1^Division of Pediatric Anesthesiology and Critical Care Medicine, The Johns Hopkins Medical Institutions, 1800 Orleans Street, Baltimore, MD 21287, USA; ^2^Division of Pediatric Critical Care, University of Virginia School of Medicine, P.O. Box 800386, Charlottesville, VA 22908, USA

## Abstract

*Introduction*. Human metapneumovirus (HMPV) is a paramyxovirus from the same subfamily as respiratory syncytial virus (RSV) and causes similar acute lower respiratory tract infection. Albuterol in the setting of acute RSV infection is controversial and has not yet been studied in HMPV. We sought to determine the frequency of albuterol use in HMPV infection and the association between albuterol administration and patient outcomes.* Methods*. We conducted a retrospective cohort study identifying all patients hospitalized in a tertiary care children's hospital with laboratory-confirmed HMPV infection between January 2010 and December 2010.* Results*. There were 207 patients included in the study; 57% had a chronic medical condition. The median hospital length of stay was 3 days. Only 31% of patients in the study had a documented wheezing history, while 69% of patients received at least one albuterol treatment. There was no difference in length of stay between patients who received albuterol and those who did not.* Conclusion*. There is a high frequency of albuterol use in children hospitalized with HMPV infection. As with RSV, evidence may not support routine use of bronchodilators in patients with acute HMPV respiratory infection. Research involving additional patient outcomes and illness severity indicators would be useful in future studies.

## 1. Introduction

Viral respiratory infections are a leading cause of illness and hospitalization in infants and children worldwide. In 2009 in the United States, more than 300,000 children were hospitalized as a result of infectious respiratory illness [1]. Children with chronic medical conditions have an increased risk of morbidity and mortality from these viral respiratory infections [2–7].

In 2001, a new virus of the Paramyxoviridae family called human metapneumovirus (HMPV) was identified [8]. The virus, a member of the same subfamily as respiratory syncytial virus (RSV), has been credited as the etiology of “non-RSV bronchiolitis” due to its overlapping seasonal distribution [9–11]. Children with HMPV typically present with signs and symptoms of upper and lower respiratory tract infection such as fever, cough, tachypnea, and wheezing [11]. In children less than five years of age, rates of hospitalization are similar between HMPV, parainfluenza, and seasonal influenza [12].

Nebulized respiratory medications, such as albuterol and racemic epinephrine, have historically been administered in children presenting with viral lower respiratory infection. We hypothesized that the use of albuterol in HMPV infection is common. Recent evidence has led the American Academy of Pediatrics to recommend against the routine use of inhaled beta-adrenergic agents, such as albuterol, in the treatment of bronchiolitis [13]. A recent Cochrane Database Systematic Review concluded that neither oxygen saturation nor length of hospitalization was improved by use of bronchodilators in bronchiolitis [14]. Another Cochrane Review concluded that repeated dosing of racemic epinephrine in hospitalized children with bronchiolitis is also not recommended [15]. While there is an abundance of data regarding the frequency of use and efficacy of beta-adrenergic agents and racemic epinephrine in children with RSV, there is a paucity of data as it applies to HMPV.

This study employed the following objectives: (1) to describe the clinical characteristics of patients admitted to the hospital with HMPV infection, (2) to determine the frequency of albuterol use in this population, and (3) to compare clinical outcomes, such as hospital length of stay (LOS), intensive care unit (ICU) LOS, supplemental oxygen use, and mechanical ventilation, between HMPV patients who did and did not receive albuterol. We hypothesized a high frequency of albuterol use in this patient population. We additionally hypothesized that there would be no relationship between the administration of albuterol and hospital LOS.

## 2. Methods and Materials

The Institutional Review Board at the Children's National Medical Center approved this study. All patients requiring admission to our institution with community-acquired HMPV infection between January and December 2010 were identified. We defined infection as the detection of HMPV from a nasopharyngeal or endotracheal specimen by polymerase chain reaction (PCR) testing obtained within the first 72 hours of admission. PCR results were available within 24 hours of specimen collection. It was the general practice of our institution during the period of study to obtain respiratory viral PCR testing in any patient admitted to the hospital with infectious respiratory symptoms.

The Microbiology Laboratory at our institution uses a multiplex respiratory viral PCR: the Qiagen Symphony RGQ platform for nucleic acid extraction and amplification and a Luminex system and test reagents for the determination of assay results. Other viruses identified with this PCR include RSV, influenza A and influenza B, adenoviruses, parainfluenza viruses 1–3, and human rhino/enteroviruses. We defined viral coinfection as the detection of other viral agents by PCR testing on the same specimen that detected HMPV.

Patients with chronic medical conditions associated with increased morbidity and mortality with viral respiratory illness were identified [2–4, 16]. These conditions included chronic pulmonary conditions, cyanotic heart disease, prematurity, immunocompromised state, neuromuscular disorders, and hemoglobinopathies. Patients with a prior history of wheezing but without a formal diagnosis of asthma were identified from the electronic medical record, including notation of albuterol in home medications and documentation of wheezing in the past medical history or in any other section of clinician charting from that concurrent admission.

Review of clinical and administrative databases and the electronic medical record was conducted by a single reviewer to establish hospital LOS, ICU LOS, use of inhaled respiratory medications (e.g., albuterol, levalbuterol, racemic epinephrine, and/or ipratropium), supplemental oxygen use, chief complaint, and need for mechanical ventilation. Use of inhaled medications was determined through review of the patient's medication administration record for the patient's entire stay. Duration of use and number of doses administered were also determined. An “albuterol trial” was defined as receipt of at least one dose of nebulized albuterol during admission and “prolonged albuterol” was defined as continued receipt beyond 24 hours of admission. Data was also collected on children identified as “albuterol responders.” These were children documented in the medical chart to clinically improve after albuterol based on a charting provider's evaluation of the patient. These providers included physicians, nurse practitioners, and respiratory therapists, and the clinical improvement was charted in varying words, including “improvement in wheezing, decreased respiratory rate, or decreased work of breathing” following a treatment. Based on admitting diagnosis in the electronic medical record, patients were also categorized as admitted with a primary respiratory or nonrespiratory complaint. Supplemental oxygen use was defined as the need for supplemental oxygen not associated with inhaled respiratory treatments. Patients were stratified based on whether or not they received treatment with albuterol. The primary outcome of interest was hospital LOS.

Continuous variables were compared using the Wilcoxon rank sum testing. Categorical variables were compared using Chi square or Fisher's exact testing as appropriate. Multivariable regression was performed to adjust for potential confounders. Type-one error was set at 0.05. We calculated that a sample size of 120 patients would be needed to detect a difference in hospital length of stay of one day between groups (albuterol versus no albuterol) to achieve 80% power employing previously published hospital LOS estimates [17]. All calculations were performed using Stata/IC 12.1 (Stata Corporation, College Station, TX).

## 3. Results

There were 207 patients included in our analysis. The median age was 16 months (interquartile range (IQR) 8–45 months) and 82 patients were female (40%). One hundred eighty-eight patients (91%) had positive specimens obtained prior to, or on the day of, hospital admission. Eighty-three percent of patients presented during the months of December to April with a peak incidence during the month of March (61 patients, 29%).

The most common diagnoses on admission were acute respiratory tract infection (70%), status asthmaticus or asthma exacerbation (14%), vaso-occlusive crisis (4%), and seizure (3%). The characteristics of patients hospitalized with community-acquired laboratory-confirmed HMPV infection are listed in Table 1.

Of the 31% of patients with a documented previous history of asthma or wheezing, 94% of them received trial of albuterol therapy during their hospitalization. 81% of those with previous asthma or wheezing also received multiple albuterol treatments. One hundred percent of patients admitted with an asthma-related diagnosis received albuterol. Of the 179 patients admitted with a nonasthma-related diagnosis, 114 patients (64%) received albuterol.

The median hospital LOS was three days (IQR 2–5 days). Forty-one patients (20%) were admitted to an ICU, either pediatric or cardiac ICU. In patients admitted to an ICU, the median ICU LOS was four days (IQR 1–10 days). One hundred forty-two patients (69%) received a trial of albuterol treatment. One hundred two patients (49%) received multiple albuterol treatments with albuterol during the first 24 hours of hospitalization. One hundred fourteen patients (55%) received supplemental oxygen. Thirteen patients (6%) required mechanical ventilation and four patients (2%) did not survive to discharge.

We compared patient characteristics and clinical factors between patients who received albuterol and those who did not receive albuterol (Table 2). There was no difference in hospital LOS between patients who received albuterol and those who did not. There was no difference in albuterol use based on need for ICU admission. Patients with a previous diagnosis of asthma and those admitted with an asthma-related diagnosis were more likely to receive albuterol (both *p* < 0.001). Patients with a nonasthma chronic medical condition were no more likely to receive albuterol than asthmatics and previously healthy patients (*p* = 0.09). Upon exclusion of asthmatics, those patients with a nonasthma chronic medical condition were more likely to receive albuterol than previously healthy children (*p* < 0.001). Patients who received albuterol were more likely to have received supplemental oxygen (*p* < 0.001). Male patients were more likely to receive albuterol (*p* = 0.03) than females, although, in our cohort, male patients were more likely to have a history of asthma (*p* = 0.01) than females. There was no correlation between age, sex, or chronic medical conditions with length of stay. Patients requiring supplemental oxygen had a longer length of stay (median 4 days versus 2 days, *p* < 0.001). Children with prior wheezing or asthma had a shorter LOS (*p* = 0.001, median 2 days versus 3 days).

Fifty-five percent of children in this study received prolonged albuterol therapy with a median duration of albuterol administration of four days (IQR 3−5 days). Patients receiving prolonged albuterol beyond 24 hours had a longer median hospital LOS (3 days versus 2 days, *p* = 0.025). This difference ceased to be significant after adjusting for supplemental oxygen use and asthma or prior wheezing. However, among the 80% of patients that did not require ICU admission, prolonged albuterol use was associated with longer hospital LOS even after adjusting for supplemental oxygen use and asthma or prior wheezing (*p* = 0.046). Prolonged albuterol use was associated with an additional 1.7 hospital days after adjusting for supplemental oxygen use and asthma or prior wheezing among the cohort not requiring ICU admission.

Ninety-eight patients (47%) received at least one other nebulized respiratory medication: ipratropium (72 patients), racemic epinephrine (27 patients), dornase alfa (2 patients), and dexamethasone (1 patient). Patients who received a secondary nebulized respiratory medication were more likely to have a chronic medical condition (*p* < 0.001). Twenty-eight (20%) of the 142 patients who received albuterol were described as albuterol responsive.

As described above, previously healthy children, who comprised 43% of our cohort, were less likely to receive any albuterol than those with asthma or other chronic medical conditions. Forty-five percent of previously healthy children received albuterol trial, with 21% receiving prolonged albuterol. There were 43 patients with viral coinfection (21%). Other viruses recovered included human rhino/enterovirus (28 patients), adenovirus (9 patients), RSV (2 patients), influenza A (2 patients), and parainfluenza 3 (2 patients). There was no association between viral coinfection and any patient characteristics or clinical outcomes, most notably receipt of albuterol or hospital length of stay.

## 4. Discussion

*Conclusion #1: The Frequency of Albuterol Trial in Children Hospitalized with HMPV Is High (69%). The Frequency of Prolonged Albuterol Use Is High in Children with HMPV (55%).* We observed a high frequency of bronchodilator use in hospitalized children in our study. Albuterol was administered to children both with and without a documented past history of wheezing or asthma. Our study observed widespread, frequent, and early (often within the first 24 hours of hospitalization) initiation of albuterol therapy to children with HMPV respiratory infection. This finding presents the question as to the reason for widespread bronchodilator use in this patient population. Clinicians may be likely to perceive some improvement in symptomatology following albuterol treatments. This may be true improvement but may also be transient due to concurrent administration of oxygen, other medication adjuncts (antipyretics, steroids, and antibiotics), and/or humidification in the setting of mask administration. Similarly, in a study of bronchodilator effects on airway reactivity in ventilated children with RSV bronchiolitis, only short term benefits were observed. In those children, acute improvement in airway reactivity, as evidenced by functional residual capacity, was suggested after albuterol treatments, but a clear translation of this improvement to long term outcomes was not observed [10]. In children with a history of chronic illness and/or wheezing, their histories often reveal home use of albuterol or prior presentations with wheezing and albuterol. This correlates with our observation that 94% of patients with a history of wheezing or asthma received albuterol. This population, however, does not fully account for the widespread use of albuterol that we observed. Only 31% of patients in the study had a documented wheezing history, while 69% of patients received albuterol.

*Conclusion #2: The Receipt of Any Albuterol Was Not Associated with Length of Stay*. We identified no difference in hospital LOS between HMPV patients who received albuterol treatment and those who did not. This finding was independent of length of albuterol treatment. In analysis of this conclusion, it is useful to consider an illness such as asthma, in which albuterol is a mainstay of treatment [18]. One study of critically ill asthmatic patients found an association between continuous albuterol and a shorter LOS as compared to intermittent albuterol administration [19]. In fact, another study looked at the specific gene known to play a role in response to beta-2 agonists such as albuterol. This study found asthmatic patients homozygous for glycine at that amino acid to have shorter ICU length of stay as compared to other genotypes [20]. Both studies suggest a role for albuterol in improvement of clinical outcomes in asthma; however, our results would not support a similar expectation in HMPV bronchiolitis. This demonstrates a difference between asthma and lower respiratory tract infection caused by HMPV. Results of our study are congruent with evidence on which the most recent AAP guidelines were based in order to conclude that the routine use of bronchodilators does not have a clear benefit in treatment of bronchiolitis [13]. Our results additionally highlight this finding in bronchiolitis caused specifically by HMPV. With attention to LOS and oxygen requirement, our findings do not support the routine use of albuterol in every patient with HMPV.

*Conclusion #3: Prolonged Albuterol Use, beyond the First 24 Hours of Hospitalization, Is Associated with Longer Hospital Length of Stay.* We observed that prolonged albuterol administration was associated with a longer hospital LOS. As the medical record does not include standardized markers of illness severity, we are unable to definitively rule out illness severity as a confounding variable of this result. One might propose that chronic illness may parallel illness severity, with those children having a longer LOS or being more likely to receive continued albuterol. We did not find an association between chronic medical conditions and length of stay, and a history of wheezing was actually found to be associated with shorter hospitalizations. A similar study in children with RSV also demonstrated an increased length of stay and duration of oxygen therapy in association with regular albuterol use [21]. As more is known about HMPV and as PCR results become more rapidly available, knowledge of the pathogen may become useful to clinicians in decisions regarding albuterol continuation beyond 24 hours.

Limitations of this study are acknowledged as this is a retrospective study where motivations to treat were not consistently clear to the researchers. Extensive chart review was conducted in order to capture those patient characteristics; however, information in electronic records is not fully standardized, nor does it guarantee completeness. Additionally, this study did not determine severity of illness in patients aside from intensive care admission and any potentially complicating chronic illness. Though we found high frequency of albuterol use in this patient population, we do not have a comparison frequency of use in all patients with respiratory illness in our institution. A patient's response to albuterol would be the ideal outcome to identify albuterol as the contributing variable, but we are limited to length of stay and prolonged albuterol administration to describe our population clinically. There is likely a difference between children experiencing an asthma exacerbation versus bronchiolitis secondary to HMPV infection, both in clinical motivators for albuterol and in disease pathophysiology. Due to the limitations of the patient chart as well as sample size, we were unable to analyze these two groups separately in regard to albuterol receipt. This distinction would be helpful in future, larger studies. There is limited information on the period of time that HMPV continues to be shed in the nasopharynx of children. Small studies and case reports suggest a period of viral shedding of one to two weeks following acute infection [22, 23]. This prolonged shedding potentially confounds a positive PCR test for HMPV, especially in setting of asymptomatic patients. Our study, however, is predominately symptomatic patients with respiratory symptoms so severe as to require hospital admission.

In conclusion, the overwhelmingly high frequency of albuterol administration in patients with HMPV infection, despite a lack of recommendations to support its practice in similar lower respiratory illnesses, is a key finding in this study. We found no clear association between hospital LOS and albuterol use in our HMPV infected patient population. In the future, research involving patients identified to have HMPV infection will be vital and useful in providing a smaller, etiology-specific cohort of patients with clear recommendations for management.

## Figures and Tables

**Table 1:** Characteristics of patients hospitalized with human metapneumovirus infection.

Characteristic	Number (%)
Female gender	80 (40%)
Age group	
<6 months	36 (17%)
6–23 months	89 (43%)
2–4 years	51 (25%)
5–12 years	24 (12%)
13–17 years	5 (2%)
18–20 years	2 (1%)
Race/ethnicity	
Black	105 (51%)
White	27 (13%)
Hispanic	66 (32%)
Other	9 (4%)
Chronic medical condition	118 (57%)
Asthma	64 (31%)
Chronic lung disease	23 (11%)
Neuromuscular disease	15 (7%)
Cyanotic heart disease	4 (2%)
Prematurity	11 (5%)
Immunosuppression/immunodeficiency (nontransplant)	7 (3%)
Hemoglobinopathy	9 (4%)

Note: 41 patients had more than one chronic medical condition that predisposed to increased risk of complications from viral illness. Those patients not included in chronic medical conditions were documented as previously healthy in chart review.

**Table 2:** Characteristics of patients hospitalized with human metapneumovirus infection stratified by receipt of albuterol.

Characteristic	Albuterol (*n* = 142)	No albuterol (*n* = 65)	*p* value
	Number (%)	Number (%)	

Age (months)	18 (IQR 9–46)	15 (IQR 6–44)	0.29
Female gender	49 (35%)	33 (51%)	0.03
CMC (nonasthma)	42 (30%)	12 (18%)	0.09
CMC (asthma)	60 (42%)	4 (6%)	<0.001
Admission diagnosis			<0.001
Asthma exacerbation	28 (20%)	0	
Acute respiratory infection	101 (71%)	43 (66%)	
Seizure	3 (2%)	3 (5%)	
Vaso-occlusive crisis	6 (4%)	2 (3%)	
Other	4 (3%)	17 (26%)	
Supplemental oxygen	94 (66%)	20 (31%)	<0.001
Hospital LOS (days)	3 (IQR 2–5)	3 (IQR 2–5)	0.18
Viral coinfection	30 (21%)	13 (20%)	0.85
Mortality	4 (3%)	0	0.31

IQR: interquartile range; CMC: chronic medical condition; LOS: length of stay.
